# Detection of Tetracycline and Colistin Residue in Broiler Meat of Siddharthanagar Municipality, Rupandehi, Nepal

**DOI:** 10.1155/vmi/4928582

**Published:** 2026-05-25

**Authors:** Narendra Prasad Bhatta, Sita Rijal, Ram Chandra Sapkota, Sirjan Bastola, Pushpa Raj Joshi, Tulsi Ram Gompo

**Affiliations:** ^1^ Institute of Agriculture and Animal Science, Tribhuvan University, Paklihawa, Rupandehi, Nepal, tribhuvan-university.edu.np; ^2^ Central Veterinary Laboratory, Department of Livestock Services, Tripureswor, Kathmandu, 46000, Nepal; ^3^ Asia-Pacific Centre for Animal Health, Melbourne Veterinary School, University of Melbourne, Parkville, Melbourne, Victoria, 3010, Australia, unimelb.edu.au

**Keywords:** antibiotic residue, antimicrobial resistance (AMR), colistin, Nepal, poultry meat, public health, Siddharthanagar

## Abstract

The widespread and unregulated use of antibiotics in poultry production has led to growing concerns over the presence of antibiotic residues in meat, contributing to the global crisis of antimicrobial resistance (AMR). In Nepal, some studies have shown that up to 62% of broiler meat samples in major cities contain antibiotic residues. However, data from international border districts such as Rupandehi remain scarce despite their high poultry trade volume and potential for informal antibiotic usage. A descriptive cross‐sectional study was conducted from August to November 2023 to detect antibiotic residues in broiler meat sold in Siddharthanagar Municipality, Rupandehi District, Nepal. A total of 82 samples (54 breast muscle and 28 liver samples) were randomly collected from retail meat shops. Each sample was tested for tetracycline and colistin sulfate residues using ELISA kits, resulting in 164 individual tests. The results revealed that the mean residue level of tetracycline was 2.86 μg/kg, while that of colistin sulfate was 20.65 μg/kg. Residue levels exceeded the mean in 28.05% of samples for tetracycline and 29.26% for colistin sulfate. Residue levels were significantly higher in liver samples compared to breast muscle (*p* < 0.05). Overall, out of 164 tests conducted, 97 tests (59.15%) were positive for antibiotic residue, while 67 tests (40.85%) were negative. This study revealed that both groups of antibiotics were within the national maximum residue limit (MRL). However, the high levels of colistin sulfate residues suggest ongoing illegal use, despite its ban in Nepal since 2019. This highlights the urgent need for a stronger regulatory framework to ensure the enforcement of existing bans on last‐resort antibiotics. Sustainable alternatives—such as probiotics, vaccines, and other nonantibiotic interventions—should be promoted to reduce antibiotic usage in poultry production.

## 1. Introduction

Poultry meat is one of the most widely consumed animal proteins worldwide (33%), after pork (36%), providing high‐quality and easily digestible protein, essential vitamins, omega‐3 fatty acids, and minerals [1–5]. The demand for poultry has increased worldwide, especially in Asian countries [6]. In Nepal, poultry meat production has grown rapidly, with annual outputs exceeding 200,000 metric tons in 2022/23 [7]. With the increasing demand for poultry meat, the use of antimicrobials is increasing among poultry producers across Nepal for growth promotion, prophylactic purposes, and therapeutic uses [8–11]. Antimicrobials have long been vital in treating infectious diseases in humans and animals and are widely used in livestock for growth enhancement and disease prevention [6, 8, 12, 13]. However, there is evidence that indiscriminate use of antimicrobials in food animals could lead to residues in food products, which might disrupt the gut microbiota leading to allergies, carcinogenicity, immunosuppression, and gastrointestinal disorders after consumption [14, 15]. Such exposure accelerates antimicrobial resistance (AMR) in humans and animal populations, as pathogens evolve to withstand treatment, resulting in prolonged illness, higher healthcare costs, and increased mortality [16–22]. Globally, AMR is projected to cause up to 10 million deaths annually by 2050 [23].

The Codex Alimentarius Commission has recommended the maximum residue limits (MRLs) of drugs that can be accepted in food (meat, milk, and eggs) for consumption [24]. The random use of antimicrobials following an insufficient withdrawal period is the cause of antimicrobial residues in animal food (meat, eggs, and milk) above the MRL [25]. Tetracycline, one of the most frequently prescribed antibiotics in Nepal, has an MRL of 200 μg/kg in muscle and 600 μg/kg in liver samples, whereas colistin—classified by the WHO [26] as a reserve drug—has an MRL of 150 μg/kg in both tissues [24, 27]. Antibiotics, including colistin, are considered last‐resort drugs for the treatment of multidrug‐resistant (MDR) organisms [28] and have been widely misused in Nepal’s poultry industry without veterinary prescription [29]. In a study in Kathmandu, the most frequently prescribed antibiotics for treatment were tylosin (47%), colistin (47%), and dual antibiotic treatments with neomycin and doxycycline (33%) [30]. Evidence of resistance is already emerging: 43.9% of *E. coli* isolates carried the mcr‐1 gene [31], 2.68% of Gram‐negative human isolates were colistin‐resistant [32], and 33.3% of chicken meat samples from four major cities contained *E. coli*, including 16 colistin‐resistant strains [33]. The previous studies mentioned above indicated the presence of colistin residues in food animals, suggesting the frequent use of colistin in the poultry sector despite its ban on use as a growth promoter in animal feed by the Department of Livestock Services (DLS) in 2016 in Nepal [19, 29]. Nine different classes of antimicrobials weighing 91,088 kg, 47,694 kg, and 45,671 kg were used in animals in Nepal in the years 2018, 2019, and 2020, respectively [21, 27]. Various studies in different parts of Nepal have shown that the prevalence of antibiotic residues ranges from 22% to 62% in chicken products such as meat and eggs, using rapid test kits [10, 34] and primarily ELISA methods [14, 35, 36].

Various test methods are used for residue detection, ranging from microbiological and immunological assays to biosensors and advanced techniques like high‐performance liquid chromatography (HPLC), gas chromatography, and liquid chromatography–tandem mass spectrometry (LC‐MS); however, they require sophisticated sample preparation, expensive machines, and trained personnel [37]. In contrast, ELISA provides high sensitivity, good selectivity, low cost, and rapid results, and unlike LC–MS/MS, it requires simpler equipment and less technical expertise, making it more practical in diagnostic laboratories in resource‐limited settings such as Nepal [35, 38]. Previous studies in Nepal have focused mainly on major cities such as Kathmandu, Chitwan, Kailali, Pokhara, and others [10, 14, 34–36], and some of these studies used rapid test kits [10, 36], which were suitable for preliminary screening but had limited quantitative accuracy. To address this gap, we conducted our study in Rupandehi, one of the largest poultry‐producing districts and the district adjacent to Northern India where informal poultry trade happens occasionally and the use of antimicrobials such as tetracycline and colistin sulfate was suspected. We selected tetracycline and colistin sulfate for our study because tetracycline is the most commonly prescribed and misused antibiotic in Nepalese poultry and colistin sulfate is a critically important last‐resort drug [24], which continues to be used illegally despite a national ban on its use in animal production. Breast muscle and liver were selected because breast meat is the most consumed portion of broiler carcasses, while the liver is the primary organ for drug metabolism and residue accumulation, making them standard matrices for residue monitoring. We used ELISA to detect residues of antibiotics because of its greater sensitivity and specificity and its ability to quantify specific antibiotic residues with validation against international standards [35, 38] and for its comparable cost to use in diagnostic veterinary laboratories. This study is expected to add to the body of evidence for residue levels in a previously unstudied region and contributes to national drug policy, while raising awareness among farmers, veterinarians, the feed industry, and consumers about the consequences of unregulated antibiotic use.

## 2. Materials and Methods

### 2.1. Study Area and Study Design

**FIGURE 1:**
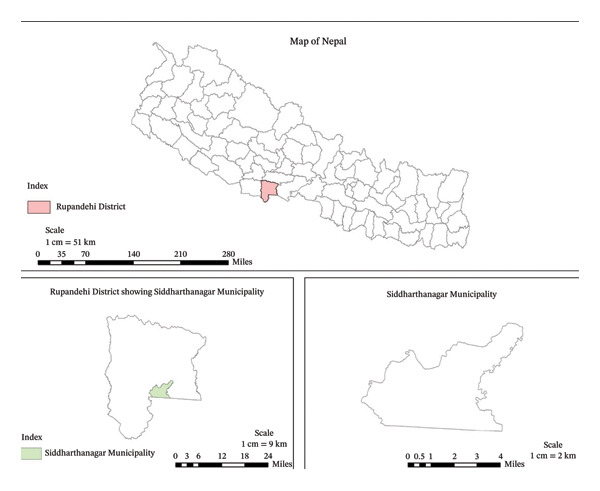
Map of Siddharthanagar Municipality (source: generated using QGIS software).

A laboratory‐based descriptive and cross‐sectional survey was conducted from August 2023 to November 2023 at Siddharthanagar Municipality, Rupandehi District, Nepal (Figure 1). The study district is an industrial hub with high poultry production potential that shares a border with Northern India, making it a high‐risk area for informal poultry trade and possible misuse of veterinary drugs in food‐producing animals.

### 2.2. Sampling

A total of 82 broiler meat samples (54 breast muscle and 28 liver samples) were randomly collected from retail outlets across 13 wards of Siddharthanagar Municipality, Rupandehi District. Municipal records for the fiscal year 2023 listed approximately 120 active poultry retailers in Siddharthanagar Municipality, which formed the basis for our sampling frame. Shops selling spoiled meat or unwilling to participate were excluded. From the remaining eligible retailers, outlets were selected using a simple random sampling method, with random numbers generated to determine shop selection. Each eligible retailer had an equal probability of being chosen, and no shop was sampled more than once. Approximately 50 g of broiler tissue was collected per outlet. The sample size was chosen to represent vendors across the municipality while remaining feasible for laboratory testing. Samples were transported in a cold chain and stored at −40°C until analysis.

### 2.3. Materials Used

Various laboratory equipment and reagents were utilized to ensure proper sample handling, preparation, and analysis. Zip‐lock plastic bags were used to collect and store the meat samples, which were transported in a cool box with ice packs and subsequently stored in a deep freezer at −40°C. Each sample was homogenized individually using either a grinder or a mortar and pestle, with all contact surfaces thoroughly sterilized between samples to prevent cross‐contamination. Micropipettes (20–200, 100–1000, and a 250‐μL multipipette) were used for sample handling, along with corresponding sterile pipette tips. Vortex mixers were used for thorough mixing, and centrifugation was performed using a standard centrifuge.

Additional materials included sample tubes, absorbent paper for use with microtiter plates, and an incubator. A digital balance with 0.01 g sensitivity was used for accurate sample weighing. Glassware such as beakers was used for solution preparation. Reagents such as distilled water, concentrated sulfuric acid (H_2_SO_4_), concentrated hydrochloric acid (HCl), sodium chloride (NaCl), and methanol were used during the extraction and sample preparation steps. ELISA plates were read at 450 nm, with a reference wavelength of 630 nm using a microtiter plate spectrophotometer (Thermo Scientific Multiskan FC).

For the detection of antibiotic residues, commercial ELISA kits were used: the tetracycline ELISA kit (Cusabio Technology LLC, China) and the colistin sulfate ELISA kit (Creative Diagnostics, USA). Each kit included specific components for quantitative residue detection. The tetracycline kit comprised a 96‐well microtiter plate coated with specific antigens, six standard solutions, HRP‐conjugate, antibody, substrate solutions A and B, stop solution, 20x wash buffer, and 10x sample extraction solution. Similarly, the colistin sulfate kit included a coated 96‐well microtiter plate, six standard solutions, enzyme conjugate and its diluent, substrate solutions (A and B), stop solution, 20x wash solution, and extraction solution.

### 2.4. Laboratory Analysis

The samples stored in the deep freezer (−40°C) were thawed overnight in the refrigerator and ground using a mortar and pestle (liver) or grinder (breast meat). The ground meat samples were treated with diluting reagents according to the instructions of the respective manufacturers for each test. The samples were then tested using quantitative ELISA assays for tetracycline and colistin sulfate residues. All test procedures were carried out at room temperature (20°C–25°C).

### 2.5. Quantitative ELISA Test

#### 2.5.1. For Tetracycline

For the quantitative detection of tetracycline residue in meat samples, the ELISA kit from Cusabio Technology LLC, China, was used [39]. Samples and reagents were prepared according to the manufacturer’s instructions provided with the kit.

#### 2.5.2. Reagent Preparation

1. Preparation of extraction solution A: 50 mL of 10x sample extraction was diluted in 450 mL of distilled water.
2. Wash buffer solution: 15 mL of 20x wash buffer was diluted into 285 mL distilled water to prepare 300 mL of 1x wash buffer.

#### 2.5.3. Sample Preparation

Approximately 1.00 ± 0.05 g of the homogenized broiler meat was weighed into a 50 mL centrifuge tube, mixed with 8 mL of Extraction Solution A, and vortexed for 3 min. The mixture was centrifuged at > 4000 rpm/min for 10 min at room temperature (20°C–25°C). A 50 μL aliquot of the supernatant was used for ELISA. A dilution factor of 8 was applied, and concentrations were determined according to the kit protocol [39].

#### 2.5.4. For Colistin Sulfate

For the quantitative detection of colistin sulfate residues, the ELISA kit from Creative Diagnostics, based on competitive ELISA technology, was used. Samples and reagents were prepared according to the manufacturer’s instructions (https://www.creative-diagnostics.com/Colistin-ELISA-Kit-3583-464.htm).

#### 2.5.5. Reagent Preparation

Solution 1 (tissue extraction): 5 mL of concentrated hydrochloric acid was diluted with deionized water to 115 mL; then, 7.5 g of sodium chloride was added and mixed completely.

Solution 2 (wash solution): Concentrated wash solution was diluted with deionized water at a 1:19 ratio.

#### 2.5.6. Sample Preparation

Approximately 1.00 ± 0.05 g of homogenized broiler meat was placed in a 50 mL polystyrene centrifuge tube with 1 mL of tissue extraction solution (Solution 1) and 2 mL of methanol. The mixture was vortexed for 2 min and centrifuged at 3000 rpm/min for 5 min at room temperature (20°C–25°C). A 50 μL aliquot of the supernatant was combined with 450 μL of kit extraction solution and vortexed for 20 s, and 50 μL of the prepared solution was used for assay. A dilution factor of 40 was applied, and concentrations were determined according to the kit protocol (https://www.creative-diagnostics.com/Colistin-ELISA-Kit-3583-464.htm).

### 2.6. Data Analysis

ELISA plates were read using Thermo Scientific Multiskan FC (SkanIt Software 4.1) at 450 nm. Optical density (OD) values were analyzed, and corresponding antibiotic residue concentrations were determined using CurveExpert software according to the manufacturer’s protocol.

#### 2.6.1. Theory Behind Using CurveExpert Software

CurveExpert software identifies the best‐fit regression model for the relationship between OD values and standard concentrations. The software selects the best regression models (e.g., logistic, rational function, and polynomial) for the given standard data, generated by the given standard concentration, while optimizing the ELISA before running the sample, and calculates the correlation coefficient (*r*) for each. The model with an *r* value closest to 1 indicates the strongest correlation and best fit. Therefore, the model with the highest *r* values is selected after several iterations by the software. Generally, for ELISA data, logistic regression models are commonly used because they effectively model the sigmoidal dose‐response relationship typical in immunoassays (https://www.cusabio.com/m-225.html#a04), as mentioned in Equation 1.

**FIGURE 2:**
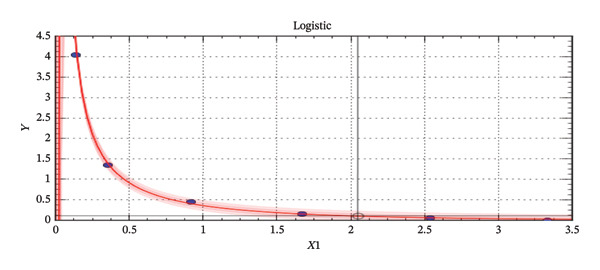
Logistic regression curve for tetracycline residue determination based on OD values.

Logistic regression model (Equation 1): *y* = *a*/(1 + *b* ∗ exp (−*cx*)) (Figure 2) (best fit generated for an antimicrobial by CurveExpert software).

“*a*” represents the upper value that the curve approaches as *x* (OD value) increases. It reflects the maximum response or signal in the assay. “*b*” is a coefficient that affects the position of the curve along the *x*‐axis, shifting the curve left or right, influencing the point at which the response starts to increase significantly. “*c*” is the slope factor or rate constant, which controls the steepness of the curve, and “*x*” is the independent variable, typically the OD value in ELISA.

**FIGURE 3:**
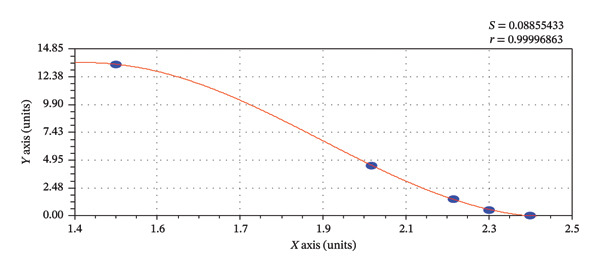
Sinusoidal fit curve for colistin sulfate residue determination based on OD values.

The absorbance values of the standards were plotted on the *y*‐axis, while the semi‐logarithmic concentrations of the tetracycline standard solution (ppb) were plotted on the *x*‐axis. The same method was followed for colistin sulfate. The resulting standard curve followed a logistic model (Figure 2) for tetracycline residue determination and a sinusoidal fit model (Figure 3) for colistin residue determination in different meat samples based on their respective OD values. The concentrations of tetracycline and colistin in each sample (ppb), obtained from the standard curve, were multiplied by their respective dilution factors (8 for tetracycline and 40 for colistin) to determine the actual concentration in the samples. The results from the ELISA of all meat samples were entered in a Microsoft Excel spreadsheet and tested for statistical significance. For the analysis of antibiotic residue presence, a two‐sample *t*‐test with unequal variances was performed to examine the association between antibiotic residues, meat samples type, and antibiotic group. A *p* value of less than 0.05 at the 95% confidence interval (CI) was considered statistically significant. Finally, tables and figures were used to present the results generated, and a graphical presentation was completed in MS Excel 2016.

For our data, we got, for the logistic model (Equation 1): *y* = *a*/(1 + *b* ∗ exp (−*cx*)) (Figure 2).

*a* = −0.426, *b* = −0.988, and *c* = −0.792.

For the sinusoidal fit (Equation 2): *y* = *a* + *b* ∗ cos (*cx* + *d*) (Figure 3).

*a* = 6.76, *b* = 6.90, *c* = 2.81, and *d* = −3.87.

## 3. Results

### 3.1. Residue Level of Tetracycline in Various Meat Samples

**FIGURE 4:**
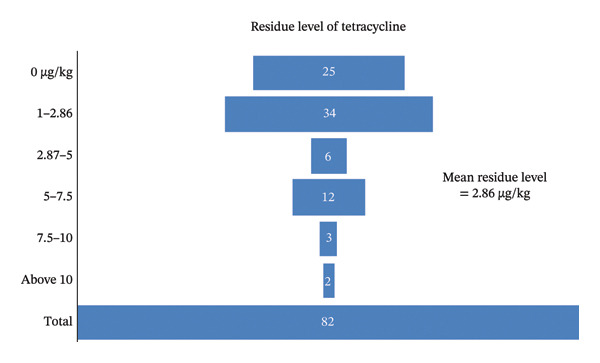
Funnel chart showing distribution of tetracycline residue levels in meat samples.

Based on logistic regression model (Equation 1), out of 82 samples, 57 (69.51%) tested positive for tetracycline residues, while 25 (30.49%) tested negative. Among the positive samples, 34 (41.46% of all samples) had residue levels below the mean value of 2.86 μg/kg, and 23 (28.05% of all samples) were above the mean. Specifically, 6 samples (7.31%) had residues between 2.87 and 5 μg/kg, 12 (14.63%) between 5 and 7.5 μg/kg, 3 (3.65%) between 7.5 and 10 μg/kg, and 2 (2.43%) above 10 μg/kg. The maximum residue detected was 17.10 μg/kg in a liver sample, and the minimum was 0 μg/kg. Results are illustrated in Figure 4.

### 3.2. Tetracycline Residues Relative to the Mean Value in Breast Muscle and Liver Samples

**FIGURE 5:**
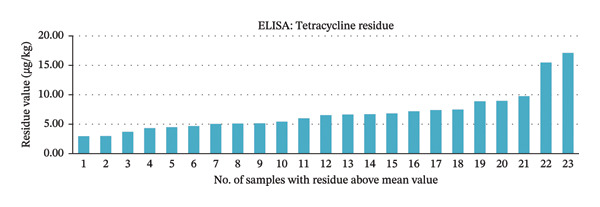
Bar graph of tetracycline residues above the mean value (2.86 μg/kg).

Among the 82 meat samples, 23 (28.05%) contained tetracycline residues above the mean residue level of 2.86 μg/kg (Figure 5). None of the samples exceeded the MRLs, which are 200 μg/kg and 600 μg/kg for muscle and liver samples, respectively. The difference in residue levels between breast muscle and liver samples was statistically significant (*p* < 0.05).

### 3.3. Residue Levels of Colistin Sulfate in Various Meat Samples

**FIGURE 6:**
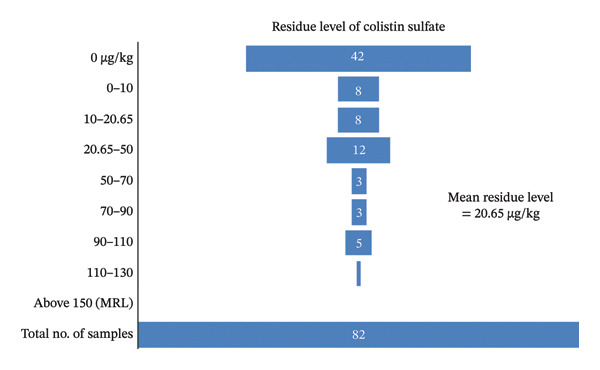
Funnel chart showing distribution of colistin sulfate residue levels in meat samples.

Based on the sinusoidal fit model (Equation 2), out of 82 samples, 40 (48.78%) tested positive for colistin sulfate residues, while 42 (51.22%) tested negative. The mean residue level among positives was 20.65 μg/kg. Of the positive samples, 8 (9.75% of all samples) had residues below 10 μg/kg, 8 (9.75%) between 10 and 20.65 μg/kg, and 12 (14.63%) between 20.65 and 50 μg/kg. Higher concentrations were observed in 3 samples (3.65%) between 50 and 70 μg/kg, 3 (3.65%) between 70 and 90 μg/kg, 5 (6.09%) between 90 and 110 μg/kg, and 1 (1.21%) between 110 and 130 μg/kg. The maximum residue detected was 126.04 μg/kg in a liver sample, and the minimum was 0 μg/kg. Results are shown in Figure 6.

### 3.4. Profile of Colistin Sulfate Residues Relative to the Mean Value in Samples

**FIGURE 7:**
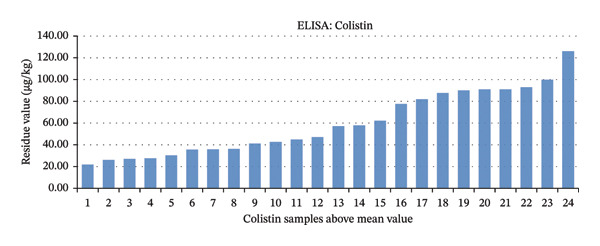
Bar graph of colistin sulfate residues above the mean value (20.65 μg/kg).

Among the 82 meat samples, 24 (29.27%) contained colistin sulfate residues above the mean value of 20.65 μg/kg (Figure 7). None of the samples exceeded the MRL, which is 150 μg/kg for both liver and muscle samples. The difference in residue levels between breast muscle and liver samples was statistically significant (*p* < 0.05).

### 3.5. Status of Single or Both Antibiotic Residues in Samples

**FIGURE 8:**
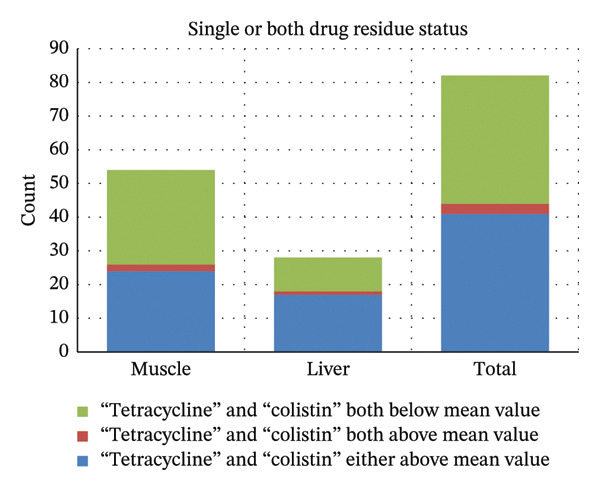
Bar graph showing profile of single or combined antibiotic residues in meat samples.

Out of 82 meat samples, 41 (50.00%) contained a single antibiotic residue (either tetracycline or colistin sulfate) above the mean value, while 3 (3.65%) contained both residues above the mean. In total, residues above the mean were detected in 47 of 164 individual tests (28.65%), indicating that at least one antibiotic residue exceeded the mean level in nearly one‐third of the assays. Results are illustrated in Figure 8.

### 3.6. Profile of Antibiotic Residues for the Meat Samples Marketed in Siddharthanagar Municipality

Out of 164 tests for tetracycline and colistin sulfate, 67 (40.85%) were negative and 97 (59.15%) were positive. Among the positives, 57 samples contained tetracycline residues (30 breast muscle and 27 liver samples), while 40 samples contained colistin sulfate residues (32 breast muscle and 8 liver samples). In total, 28 breast muscle samples and 19 liver samples had residues above the mean value for at least one antibiotic. Overall, 47 of 164 tests showed residue levels above the mean. A tabular representation is provided in Tables 1 and 2.

**TABLE 1:** Distribution of tetracycline and colistin sulfate residues in broiler meat samples (*n* = 82, total assays = 164).

Antibiotics	Total sample tested	No. of samples with residues (positive samples)		No. of samples with no residues (negative samples)
		Breast muscle (*n* = 54)	Liver (*n* = 28)	
Tetracycline	82	30	27	25
Colistin sulfate	82	32	8	42
Total	164	97/164 (59.15%)		67/164 (40.85%)

*Note:* Percentages are calculated based on total assays (*n* = 164). Each sample (*n* = 82) was tested for two antibiotics.

**TABLE 2:** Mean residue levels of tetracycline and colistin sulfate in broiler meat samples and no. of samples exceeding mean residue or national maximum residue limit (MRL).

Antibiotics	Sample mean residue level (*μ*g/kg)	No. of samples above mean residue level		Total no. of samples above mean residue	Total no. of samples above national MRL
		Breast muscle (*n* = 54)	Liver (*n* = 28)		
Tetracycline	2.86	7	16	23	0
Colistin sulfate	20.65	21	3	24	0
Total		28	19	47/164	

*Note:* “Above mean” are calculated as number of samples above mean residue.

## 4. Discussion

This study confirmed the presence of antibiotic residues in the broiler meat samples. Analysis of 82 broiler meat samples (54 breast muscle and 28 liver samples) from Siddharthanagar Municipality revealed tetracycline residues in 57 samples (69.51%) and colistin sulfate residues in 40 samples (48.78%). Although tetracycline was more prevalent, colistin sulfate residues were detected at higher concentrations. The relatively elevated levels of tetracycline and colistin sulfate in liver samples reflect the role of the liver in drug metabolism and detoxification, a pattern consistently reported in previous studies [13, 35, 40]. The mean residue levels were 2.86 μg/kg for tetracycline and 20.65 μg/kg for colistin sulfate. These findings align closely with Gompo et al. [35], who reported a tetracycline residue level of 1.44 μg/kg. None of the samples exceeded the MRL for either antibiotic. For tetracycline, this suggests partial compliance with withdrawal periods and drug use guidelines, although the widespread detection of sub‐MRL residues raises concerns about chronic low‐level exposure and its contribution to AMR [18–20]. In contrast, the detection of colistin sulfate residues is more alarming. Colistin is a critically important last‐resort antibiotic reserved for treating MDR infections in humans, and its use in food animals has been banned in Nepal. Even sub‐MRL levels of colistin sulfate indicate ongoing misuse and weak enforcement of existing regulations [30, 31, 41]. Continued misuse in poultry production accelerates the emergence of resistant strains, posing a serious public health risk. Out of 164 assays tested, 97 (59.15%) were positive for antibiotic residues. A similar prevalence of 92 (62%) was reported by Prajapati et al. [14] in Kathmandu, Kaski, and Chitwan, while Maharjan et al. [10], Chiranjibi et al. [40], and Raut et al. [34] reported lower levels, with prevalence of 150 (28.25%), 132 (30.81%), and 55 (22.77%), respectively. These variations likely reflect differences in sampling, geographic coverage, and the number of antibiotics tested but collectively confirm that antimicrobial residues are widespread in broiler meat across Nepal.

The illegal import of colistin sulfate from China and India has been reported, and it is commonly used in poultry production as a growth promoter [29]. Our findings confirm its presence in marketed broiler meat, consistent with earlier reports of misuse in Nepal. Studies by Joshi et al. [33], Bista et al. [31], and Paudel et al. [32] have already documented the emergence of colistin‐resistant bacteria, demonstrating that a drug intended as a last‐resort therapy is rapidly losing its effectiveness due to resistance development. This resistance is largely driven by farmers who use colistin sulfate indiscriminately, often without veterinary prescription, making it their first choice of treatment [29]. Although such practices may lead to modest weight gain in chicken, they also risk introducing antibiotic residues into the human food chain, negatively affecting public health [42]. Antibiotic residues in food products pose several health risks, even when below MRLs. Chronic exposure has been linked to allergic reactions, disruption of gut microbiota, and increased risk of AMR, which compromises the effectiveness of critical drugs in human medicine [16, 38, 43, 44].

ELISA remains widely used for residue detection in meat and milk due to its high sensitivity, specificity, affordability, and practicality in resource‐limited settings [14, 34, 35]. In our study, OD values were converted to concentrations using CurveExpert software, which selected the best‐fit curve for each antibiotic. A logistic model was applied for tetracycline, consistent with Gompo et al. [35], while a sinusoidal model was used for colistin sulfate, reflecting their distinct response patterns and improving quantification reliability. Comparative studies from other countries show that while ELISA is suitable for routine screening, advanced techniques such as HPLC and LC–MS/MS provide greater precision and wider analyte detection. For instance, Yuan et al. [45] demonstrated that HPLC provides more accurate quantification of ciprofloxacin residues in pork compared to ELISA, while Chan et al. [46] developed a universal LC–MS/MS method capable of detecting multiple antibiotic residues with high sensitivity and specificity. However, these methods require costly instrumentation and technical expertise, limiting their feasibility in resource‐constrained settings like Nepal. In this context, ELISA remains a practical and reliable tool for surveillance.

Our study indicates that antibiotic residues persist in marketed broiler meat, likely because farmers may be uninformed about withdrawal periods and their implications for human health. Differences in findings across studies may be due to the variations in sampling, sampling locations, methodology used, the number of antibiotics tested, and test kits used. To the best of our knowledge, no prior studies have been conducted in this area or nearby locations, as indicated by the available published data. These findings therefore provide important baseline evidence for a previously unstudied region.

This study, however, has some limitations. It was a pilot survey at Siddharthanagar Municipality which may not represent broader national patterns. While ELISA is practical in resource‐limited settings such as Nepal, it has lower precision compared to advanced techniques such as HPLC or LC–MS/MS. The cross‐sectional design also provides only a snapshot in time. Future studies should expand the geographic scope, increase sample size, and include a wider panel of antibiotics.

## 5. Conclusion

This study highlights the presence of antibiotic residues in marketed broiler meat samples. Notably, the detection of colistin sulfate residues—despite its official ban in Nepal since 2019—indicates ongoing misuse and gaps in enforcement. Our results show that 69.52% of the samples contained tetracycline residues, while 48.78% tested positive for colistin sulfate. The highest concentrations were found in liver samples, consistent with the organ’s role in drug metabolism and detoxification. Although none of the samples exceeded the national MRL, the widespread presence of residues below MRL (sub‐MRL) is concerning, as chronic exposure may have implications for antimicrobial resistance (AMR). These findings call for integrated awareness programs targeting farmers, veterinarians, and policymakers to promote responsible antimicrobial use in food animals. Eliminating the misuse of last‐resort drugs such as colistin sulfate is critical for safeguarding public health. Immediate and coordinated action is essential to reduce antibiotic residues in food products and strengthen national antimicrobial stewardship strategies.

## Author Contributions

Dr. Narendra Prasad Bhatta conceived the study, conducted field sampling, performed laboratory testing, analyzed the data, and led the writing and revision of the manuscript.

Dr. Sita Rijal supported the study design and contributed to laboratory testing.

Dr. Ram Chandra Sapkota coordinated laboratory logistics and managed the procurement of necessary test kits.

Dr. Sirjan Bastola assisted with sample collection and contributed to manuscript revision.

Dr. Tulsi Ram Gompo performed statistical analysis and contributed extensively to manuscript revision.

Dr. Pushpa Raj Joshi conducted field sampling and contributed to manuscript revision.

## Funding

No funding was received for this manuscript.

## Disclosure

All authors approved the final version of the manuscript for submission.

## Conflicts of Interest

The authors declare no conflicts of interest.

## Data Availability

Research data are not shared.
